# Diethylcarbamazine and Non-Diethylcarbamazine Related Bancroftian Granuloma: An Immunohistochemical Study of Eosinophil Toxic Proteins

**Published:** 2010-06

**Authors:** Jose Figueredo-Silva, Carmelita Cavalcanti, Luciano Tavares Montenegro, Joaquim Norões, Gerusa Dreyer

**Affiliations:** 1 *Núcleo de Ensino e Pesquisa em Patologia, Faculdade de Ciências Médicas, Universidade Estadual do Piauí, Piauí, Brazil;*; 2 *Laboratório de Imunopatologia Keizo Asami (LIKA), Universidade Federal de Pernambuco, Pernambuco, Brazil;*; 3 *Departamento de Patologia, Centro de Ciências da Saúde, Universidade Federal de Pernambuco, Pernambuco, Brazil;*; 4 *Serviço de Urologia, Departamento de Cirurgia, Centro de Ciências da Saúde, Universidade Federal de Pernambuco, Pernambuco, Brazil;*; 5 *Núcleo de Ensino Pesquisa e Assistência em Filariose (NEPAF), Hospital das Clínicas, Universidade Federal de Pernambuco, Pernambuco, Brazil;*; 6 *Centro de Pesquisas Aggeu Magalhães (CPqAM), Fundação Oswaldo Cruz (FIOCRUZ), Pernambuco, Brazil;*; 7 *NGO Amaury Coutinho Rua Conselheiro Portela, 665, Sala 120, Espinheiro, Recife, 50670-900, Pernambuco, Brazil*

**Keywords:** bancroftian filariasis, eosinophils, eosinophil cationic protein, eosinophil peroxidase, filarial granuloma, immunohistochemistry, lymphatic fiariasis, major basic protein, *Wuchereria bancrofti*

## Abstract

It has been suggested, mostly using *in vitro* experiments, that defenses against parasites involve mainly activated eosinophils and their toxic proteins, such as major basic protein (MBP), eosinophil cationic protein (ECP) and eosinophil peroxidase (EPO). Eosinophil degranulation has been described around degenerating onchocercal microfilariae in patients treated with diethylcarbamazine (DEC). In bancroftian filariasis, traditional histopathologic studies have shown remarkable numbers of eosinophils in granulomatous lesions associated with both DEC-induced and spontaneous death of adult *Wuchereria bancrofti* parasites. No immunohistochemical study targeting eosinophil degranulation has been previously performed in these granulomas, which are found mainly within intrascrotal lymphatic vessels. This investigation was undertaken in 22 (12 DEC-treated and 10 untreated) male patients in order to determine the immunohistochemical expressions of MBP, EPO and ECP in bancofitian granulomas, using the indirect method. Stained intact esosinophils, as well as granular, extra-cellular material positive for all three proteins, were found in all granulomas. The immunohistochemical patterns were similar in both DEC-treated and untreated cases, irrespective of microfilaremia, blood eosinophilia, and granuloma age. Positive intact cells were observed mostly at the periphery of the granulomas, whereas granular material predominated in central areas around dead or degenerating parasites. These results indicate that eosinophils accumulate in the granulomas and degranulate preferentially in close proximity to degenerating or dead adult parasites. In bancroftian granulomas, influx and degranulation of eosinophils are considered a consequence of parasite death, rather than its cause.

## INTRODUCTION

The association between parasitic infections and eosinophilia in the blood and tissues of the host has been known for more than a century and is well-documented, being helminths the most common cause of blood eosinophilia in global terms (1). The exact role of eosinophils in host protection is still debated (2), but they seem primarily to be potent effector cells involved in the defense against infective larval stages of parasitic helminths. In contrast, living adult worms appear to be resistant to eosinophil attack (3). Since the *in vivo* mechanisms of action of human eosinophils have been difficult to establish, information has being gathered mostly from unnatural hosts as experimental models or *in vitro* studies. For instance, *in vitro* investigations have shown that eosinophils operate via ADCC (antibody-dependent cytotoxicity) directed against schistosomula (4) and microfilariae of *Onchocerca volvulus* (5).

Although ADCC, appears well suited for killing parasites too large to be phagocytized, it has been difficult to prove that it actually occurs *in vivo* (6). In fact, data supporting eosinophil function *in vivo* are mainly indirect since the evidence refers to interferences in interleukin-dependent responses (7). Several studies have also shown that eosinophil-derived cationic proteins, such as the major basic protein (MBP), the eosinophil cationic protein (ECP), and the eosinophil peroxidase (EPO) are particularly involved in cytotoxic activity (8-11). Nevertheless, these highly toxic eosinophil proteins are responsible for a considerable amount of the host inflammatory pathology that accompanies helminth infections (12).

Previous histological studies have shown close associations between tissue eosinophils and adult *Wuchereria bancrofti* parasites, damaged or dead, both during the course of diethylcarbamazine (DEC) treatment and naturally (13-19). Segments of coiled parasites in different stages of disruption are usually encircled by a granulomatous inflammatory reaction with remarkable numbers of eosinophils, as described in detail elsewhere (20). In such scenarios, eosinophil degranulation is expected to occur, but no studies have specifically investigated this. In men, spontaneous or drug-induced adult *W. bancrofti* parasite death is followed by the development of a palpable nodule, most commonly localized in intrascrotal lymphatic vessels (21, 22). The present study was undertaken to determine the immunohistochemical expression of MBP, EPO and ECP in granulomatous lesions in scrotal nodules removed from DEC-treated and non-treated patients.

## MATERIAL AND METHODS

### Tissue specimens

This retrospective study was conducted at the Center for Teaching, Research and Tertiary Referral for Bancroftian Filariasis (NEPAF) and the Laboratory of Immunopathology Keizo Asami-LIKA, both at Federal University of Pernambuco in Recife, Brazil. Tissue specimens for this study were randomly selected from the filariasis histopathology tissue bank. The material consisted of excised nodules that had been detected by physical examination of the intrascrotal contents; they were obtained during previous trials described elsewhere (17, 18, 23-26) and from patients participating in a diagnostic protocol used at NEPAF (J. Norões, unpublished). Before nodule excision, all patients signed an informed consent form. The study was conducted in accordance with the principles of the Declaration of Helsinki and the guidelines on Good Clinical Practice. Granuloma age, which could be determined only for patients treated with DEC, was defined as the interval between DEC intake and the day of excision biopsy. The DEC oral dose was 6mg/kg/day, given as a single dose or as a 12 day course.

### Microfilaria counts

The microfilaria (Mf) density was obtained from venous blood collected between 23:00 and 01:00 o’clock and filtered through a 3μm polycarbonate membrane, stained, and examined by microscopy. Amicrofilaremia was defined as having no Mf detected in 11 mL of blood (if no Mf was detected in one mL, an additional 10 mL was obtained and examined within 7-15 days) (24). In treated patients the microfilaria density was investigated before DEC intake. In the untreated patients the first blood sample was collected one to three days after palpation of the nodule by the examining physician.

### Blood absolute eosinophil counts

The peripheral eosinophil concentration was measured by Neubauer counting chamber. All patients receiving DEC blood eosinophilia was avaluated before and between 7 and 14 days after the first dose of DEC intake as described elsewhere (27). In patients with spontaneous nodules (i.e., those who had not received DEC), the eosinophil counts were measured when the nodule was first palpated by the examining physician and 7 to 14 days later.

### Histopathology

The surgical specimens, fixed in buffered 10% formalin, were elastic, white-grayish, and either chord-like (varying from 2.5 × 0.5 cm to 4.5 × 0.4 cm) or ovoid (0.5 to 2.5 cm in its largest diameter). Several transverse slices from each specimen were routinely processed, and 5 μm-thick sections were stained with hematoxylin-eosin and Masson’s trichrome, as previously described (19). Images were obtained with an Olympus CX31 microscope and an Olympus digital camera C-7070 (Tokyo, Japan).

### Immunohistochemistry for major basic protein (MBP), eosinophilic peroxidase (EPO) and eosinophilic cationic protein (ECP)

Immunohistochemical staining was conducted using the standard indirect method. A set of six de-paraffinized 5 μm-thick sections of the same material used for the histopathological study was subjected to microwave for 20 minutes at maximum power as an antigen retrieval method. The endogenous peroxidases were blocked with a methanol solution containing 0.03% hydrogen peroxide and 1% sodium azide for 20 minutes. Tissue sections were then incubated overnight at 4°C with either normal swine serum (Dako Corporation, Carpinteria, CA) as a negative control, or with primary high-affinity chromatrography-purified rabbit IgG antibodies to MBP, EPO, and ECP. Goat high-affinity anti-Fc-rabbit IgG conjugated to peroxidase (Jackson Immunoresearch Laboratories, Inc., West Grove, PA) was applied to the tissues for 60 minutes at 37°C. The slides were developed using diaminobenzidine and counterstained with 10% Harris hematoxylin. After each treatment stage and incubation, the slides were washed in phosphate buffered saline. Brown staining was considered as an expression of immunoreactivity to MBP, EPO, and ECP. Interpretation of immunohistochemical results included both the location of the positive reaction and the staining pattern: intact, well-defined cells versus extra-cellular granular staining. The presence of extra-cellular positive granular material was interpreted as evidence of eosinophil degranulation. It was beyond to the scope of the study to assess quantitatively the immunohistochemical reaction for each protein studied.

### Statistics

Descriptive statistics were used in the analysis of the data (Excel 2002). Pre and post eosinophil counts were log-transformed. Means of log-transformed counts were compared using the Student t test for paired samples.

## RESULTS

### Patients

The clinical, parasitological and antifilarial treatment schedules of the patients are shown in Table 1. Peripheral blood eosinophilia in relation to Mf counts and antifilarial treatment are shown in Table 2.

**Table 1:** Clinical and parasitological features and antifilarial treatment doses of the patients

Characteristics	Total	DEC related	Not-DEC related

Number of patients (%)	22	12	10
Mean age (range), years	22.5(15-34)	22.6(19-34)	21.4(15-32)
DEC dose			
Single dose		5	NA
12-day course		7	NA
Granuloma age (range), days	30.8 (7-90)	30.8 (7-90)	NA^a^
Site of the nodule			
Right		7	5
Left		5	5
Spermatic cord		11	9
Infratesticular		1	0
Paratesticular		0	1
Geometric mean Mf density (range)			
per 1 mL of blood	34.3(0-4128)	90.7(0-4128)	9.4(0-1765)
Number of patients			
Mf+/1mL	10	6	4
Mf-/11mL	1^b^	0	1
Mf+/11mL	11	6	5

a It was not possible to provide the granuloma age, since the patients did not know when the nodule appeared (by self physical examination or by discomfort);

b 0.2 Mf/Ml.

**Table 2:** Microfilarial density and eosinophil counts in peripheral blood of treated and untreated patients

Relation to DEC treatment			Geometric mean (range)			

	Mf density	No. of patients	Mf density/mL	Eosinophils/mm^3^		p value^c^
				Pre	Post	

**DEC-related**	<100/mL	5^a^	6.5(0-65)	360.3(150-800)	352.4(175-850)	0.799
	>100/mL	7	597.4(123-4128)	303.6(50-600)	1205.4(625-3800)	0.015
**Not DEC-related ±**	<100/m	7^b^	0.8(0-0.2)	280.2(75-1650)	267.0(50-1700)	0.479
	>100/m	3	833.5(243-1765)	362.9(300-425)	689.0(475-950)	0.167
**All patients**		22	27.2(0-4128)	315.3(50-1650)	522.8(50-3800)	0.015

a Two were Mf negative;

b six were Mf negative;

c Student t test (comparing log geometric means: post versus pre observations points); ± Pre and post indicate eosinophil counts measured when the nodule was first palpated by the examining physician and 7-14 days later, respectively.

### Histopathology

**Figure 1:**
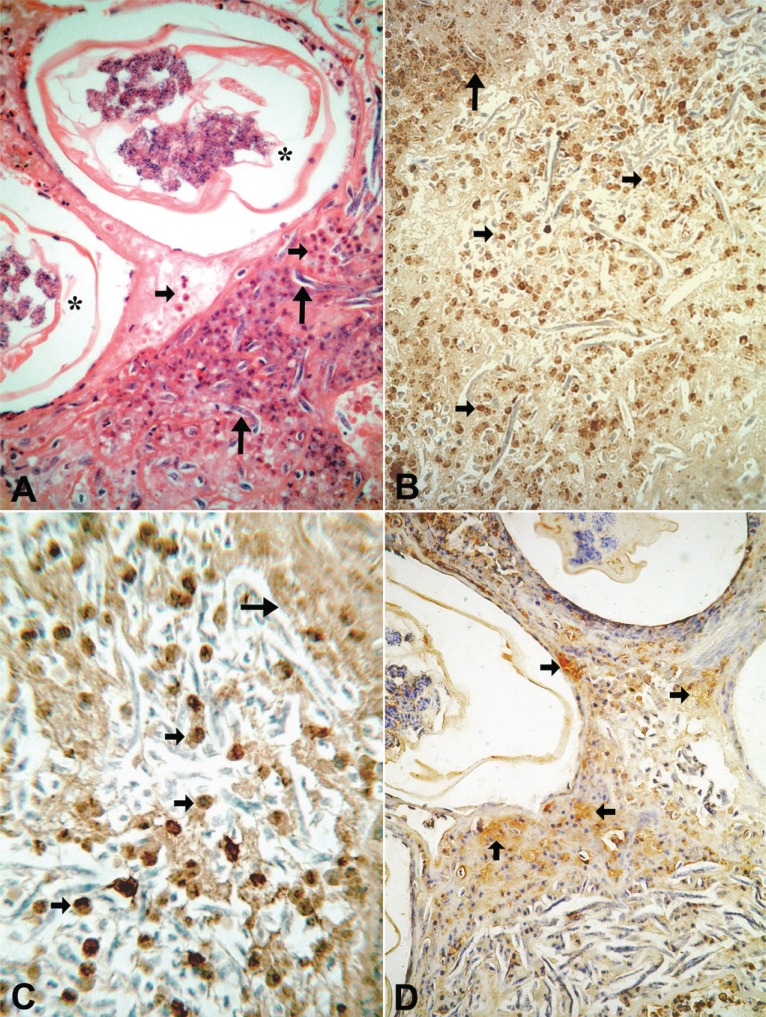
Twenty-one year-old microfilaremic (243 Mf/mL) treated patient (diethylcarbamazine 6mg/kg/day/12days) with a nodule obtained 7 days after the beginning DEC. (A) Transverse sections of degenerating adult worm (*) enclosed by fibrin-like material containing free Mf (large arrows) and inflammatory cells - mainly eosinophils (arrows) - without a granulomatous pattern (H & E. 400×). (B) Positive immunostaining for major basic protein showing large numbers of intact eosinophils (small arrows) and extracellular granular material surrounding degenerating free Mf (arrow) (400×). (C) The same immune pattern - intact cells (small arrows) and granular material (arrow) - is observed also for eosinophilic cationic protein, 600×. (D) Granular material (arrows) can be easily recognized as well as with eosinophil peroxidase staining, 400×.

**Figure 2:**
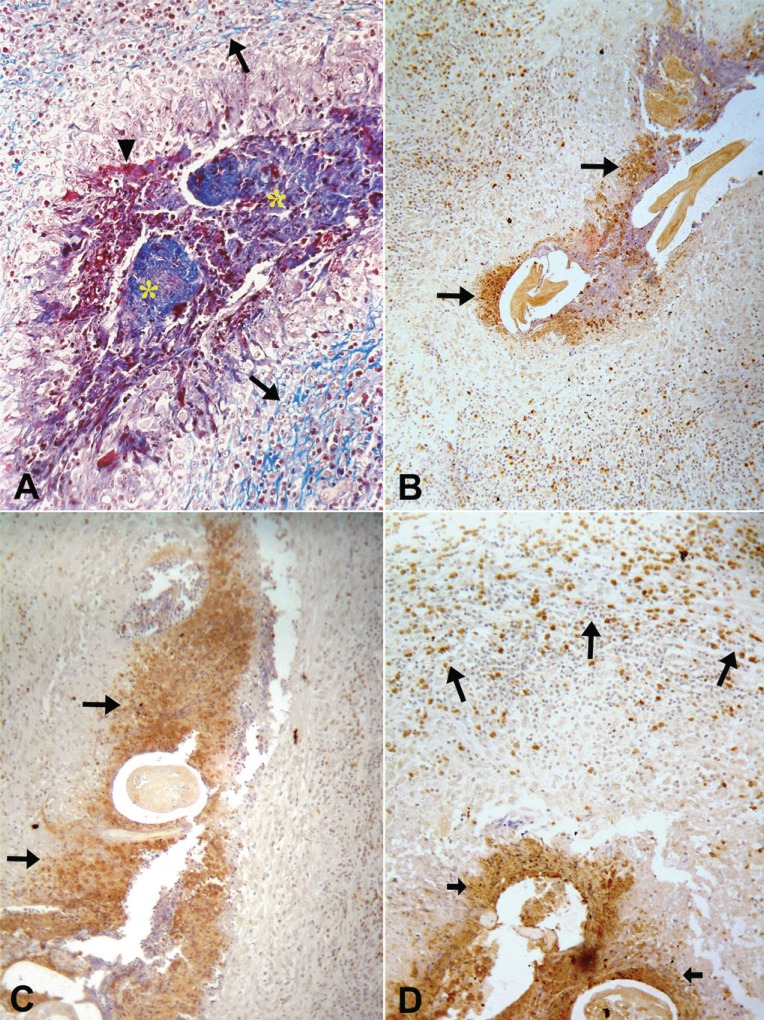
Twenty-five year-old microfilaremic (1,350 Mf/mL) untreated patient with a nodule with unknown time of evolution. (A) A granulomatous inflammatory reaction around desintegrating adult worms (*) is observed; a rim of collagen deposition is seen at the periphery (arrows). Necrotic area is seen around the degenerated worms (arrow head). Manson trichome, 400x. Granular, extracellular positive material for major basic protein (B), eosinophil cationic protein (C) is condensed around dead adult worms (arrows) (100×). (D) Granular material stained for eosinophil peroxidase (small arrows) is also detected around the worms; intact marked cells predominate at periphery of the granuloma (arrows), 400×.

**Figure 3:**
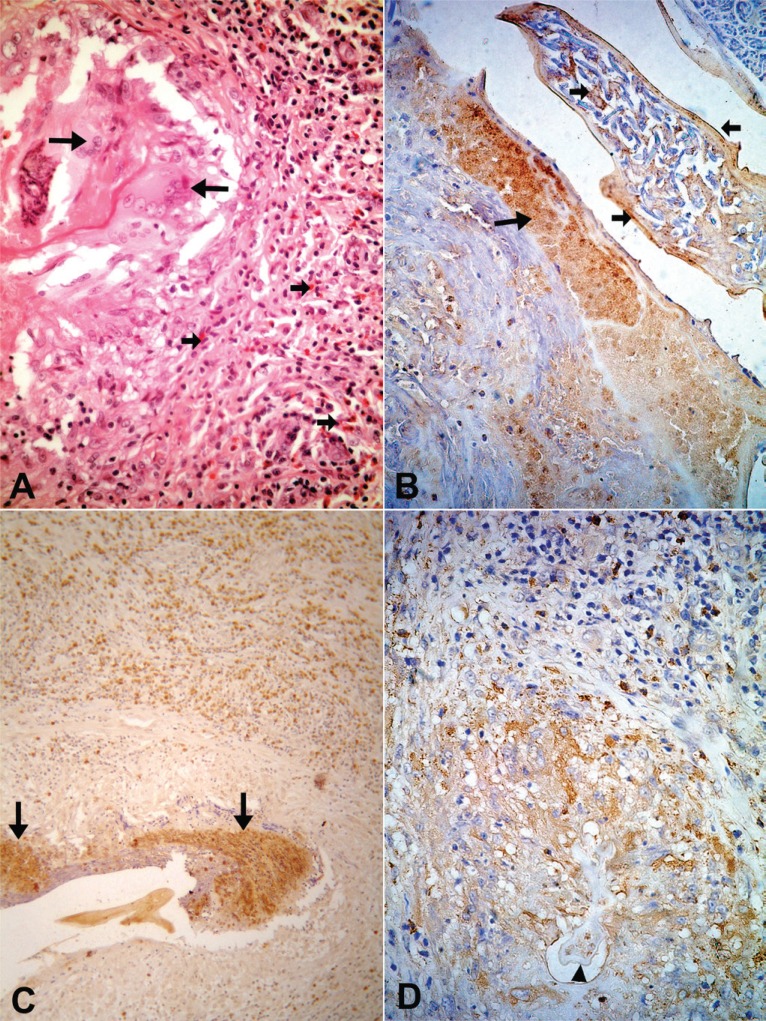
Nineteen year-old microfilaremic (124 Mf/mL) treated patient (diethylcarbamazine 6mg/kg single dose) with a nodule obtained 90 days after treatment. (A) A granulomatous response rich in eosinophils (small arrows) is observed around barely visible remnants of adult worm; the altered cuticle is seen as an eosinophilic line circumscribed by giant cells (arrows). H & E, 400×. (B) A tangential section of an adult female worm with abundant granular material positive for major basic protein (large arrow); the same material is also covering the cuticle and among intrauterine microfilariae (arrows), 400×. (C) Positive reaction is observed for eosinophilic cationic protein around degenerated worm (arrows), 100×. (D). Granular, extra-cellular material stained for eosinophilic peroxidase is present surrounding the remnants of parasite (arrow head), while intact brown, marked cells are detected at the periphery of the granuloma, 400×.

The tissue responses to the adult worm have been described in detail elsewhere (20). Briefly, the specimens consisted of lymphatic vessel segments with variable degrees of dilation. The vessels contained adult *W. bancrofti* in different stages of degeneration; progressing and healing lesions coexisted in the same tissue section. In all cases but one, a granulomatous response was seen around the damaged worms. The non-granulomatous pattern was observed in a patient seven days after beginning a 12-day course of DEC. In this case, a considerably dilated segment of the lymphatic showed cross-sections of adult worms with early signs of degeneration such as deformity and collapse of internal structures. Eosinophils, macrophages and lymphocytes infiltrated the vessel wall diffusely and were seen also entrapped in a fibrin-like material in the lumen, together with numerous free Mf (Fig. 1A). In all remaining treated and untreated cases, the lymphatic lumens were partially occluded by granulomas surrounding cross sections of dead adult worms in distinct stages of disintegration; the granulomas were composed of macrophages, lymphocytes, plasma cells and large numbers of eosinophils. In some granulomas, the damaged worms were circumscribed by a necrotic, acidophilic, granular material (Fig. 2A). The number of multinucleated giant cells and peripheral concentric fibrosis were accentuated in the more advanced cases, irrespective of antifilarial treatment and microfilaremia status. Eosinophils were present in all cases, not only within the granuloma and interspersed with collagen fibers, but also spreading into the outlying areas, even in one case 90 days after DEC treatment; in this case, a segment of damage cuticle was surrounded by giant cells (Fig. 3A). In one case, 19 days after a single DEC dose, calcified and non-calcified segments of dead parasites coexisted in the same section. Neutrophils and microabscesses formation were not observed. In 18 cases, the adult worm gender could be recognized. In 17 cases only females were found, and in one case both male and female worms were present. All females were pregnant. In the remaining four cases, worm gender could not be established due to the advanced stage of disintegration. Eleven patients were Mf-negative in 11 mL of blood (Table 1) and the tissue specimens in seven of these showed free and/or intrauterine microfilariae.

### Immunohistochemistry

**Figure 4:**
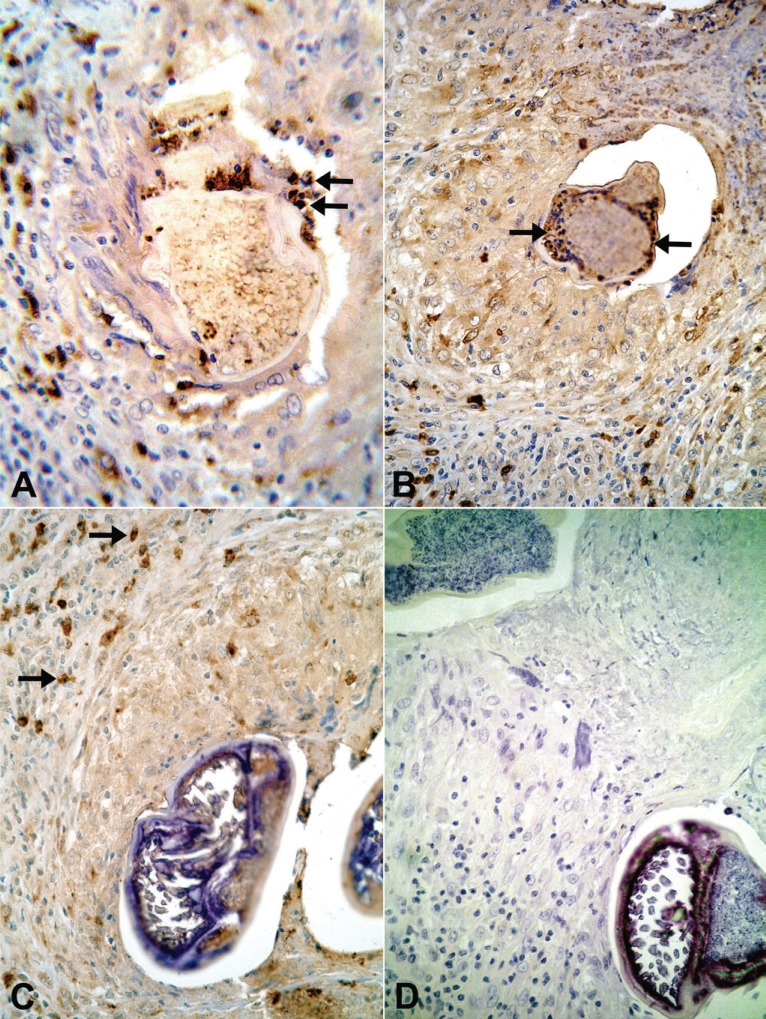
(A) Twenty-one year-old amicrofilaremic treated patient (diethylcarbamazine 6mg/kg/day/12 days) with a nodule obtained 10 days after beginning treatment. Intact cells marked for major basic protein are attached on parasite surface (arrows), 600×. (B, C and D) Nineteen year-old microfilaremic (1400 Mf/mL) untreated patient with a nodule with unknown time of evolution. (B) A collar of intact cells marked for eosinophilic peroxidase is observed on parasite surface (arrows), 400x. (C) Intact cells stained for eosinophilic cationic protein are present at the periphery of the granuloma (arrows); no positive cell is attached to the calcified wall of the parasite; only little residual amount of brown granular positive material is seen inside the parasite, 400×. (D) A control slide showed no immune staining for major basic protein. (Harris’ hematoxilyn, 400×).

In all tissue sections, intact eosinophils and granular, extra-cellular material were stained for the three proteins studied. In both DEC-treated and untreated cases, the topographic distribution of labeled cells and extra-cellular granular material was similar. In the patient who received seven days of DEC treatment and did not have a well-established granumomatous reaction, stained intact cells and extra cellular granular material were abundant in the fibrin-like material around apparently degenerated extra-uterine Mf, localized within the lymphatic lumen (Fig. 1B, C, D). In all remaining cases, numerous labeled intact cells were present in the granulomas, running in concentric layers between other unmarked cellular components of the inflammatory response, and also extending to the peripheral tissues. Granular stained material was detected mainly around damaged adult worms (Fig. 2B, C, D), corresponding to necrotic area observed in conventional histopatology (Fig. 2A). In several granulomas, worms in different stages of degradation coexisted. This was particularly exemplified in the nodule shown in Figure 3; granular stained material was detected on the surface of intrauterine Mf. Even in the specimen collected 90 days after treatment (Fig. 3B), adult worms in advanced stage of disintegration were also found (Fig. 3C, D). In two cases (treated and non-treated), intact stained cells were found in the very act of attachment to the surface of dead parasites (Fig. 4A, B). Cellular and extra-cellular positive immunohistochemical staining for the three eosinophil proteins was scarce or even absent in relation to calcified worms segments, although intact cells were still present at the periphery of the granulomas (Fig. 4C). None of the control slides revealed any imunnohistochemical staining for MBP (Fig. 4D), EPO, or ECP.

## DISCUSSION

In our study, all three toxic proteins were localized in the same areas of the granuloma in both DEC-treated and untreated cases, irrespective of microfilaremia and blood eosinophilia, and granuloma age. These findings seem to indicate that these parameters do not influence the role of eosinophils in the localized inflammatory process associated with the death of adult worms. The presence of labeled cells and granular, extra-cellular material suggests that eosinophils have accumulated and degranulated preferentially in close proximity to the adult parasites which are centrally located in the granulomas. Since eosinophils represent a hallmark of the inflammatory granulomatous reaction to degenerating adult *W. bancrofti*, it would be surprising if they did not degranulate. Indeed, it is believed that the acidophilic necrotic material observed by conventional histology (13-19) around degenerating parasites is composed, at least in part, of eosinophil-derived toxic granular material.

Positive immunohistochemical staining was seen in intact eosinophils and in extracelluar granular material around intrauterine degenerating Mf. It is likely that eosinophils reached the Mf through a disrupted wall of the adult female, a finding suggested by the concomitant presence of Mf outside the parasite. One could speculate that the eosinophil influx and degranulation might be enhanced by DEC-damaged Mf. In fact, in lymph node (28) and skin (29, 30) material from patients with onchocerciasis, it has been demonstrated that eosinophils are found degranulating at the surface of Mf soon after DEC treatment. Of interest, we recently observed a female:male prevalence ratio of 4.5:1 in granulomatous tissues material (25); all females in the present series were pregnant.

Bancroftian adult filarial worms probably die from a variety of causes, involving both parasite- and host-related factors and drugs such as DEC; however, the exact pathways leading to their death are largely unexplained (31). Whatever the cause, there are several lines of evidence indicating that the presence of eosinophils and their toxic products in tissues harboring degenerating and dead adult worms is secondary to the parasite death rather than its cause. First, although our cases were selected randomly, the study included nodules obtained from patients with and without antifilarial treatment, and with a range of microfilaremia and pre-treatment blood eosinophilia levels. Our study design allowed at least two different mechanisms of adult parasite death: DEC-related and natural contidions. The non-DEC related nodules in essence controlled for any interference of DEC on eosinophil homeostasis in microfilaremic patients such as recruitment, activation and degranulation (32). Irrespective of the clinical, parasitological and chemotherapeutic category of the patients, all the granulomas showed a similar immumohistochemical pattern for all three eosinophil proteins, making it unlikely that the death of adult *W. bancrofti* had been initiated by eosinophil-dependent or related mechanisms.

Second, absence of eosinophils in tissues containing living W. bancroti adult worms, despite peripheral blood hypereosinophilia due to concomitant intestinal helminthiases, has been reported (33). Third, post-treatment blood eosinophilia is a regular feature in response to ivermectin – a potent micro-filaricidal drug without macrofilaricidal effects – in essentially all microfilaremic patients regardless of the doses. The magnitude and kinetics of blood eosinophilia are proportional to pre-treatment Mf levels; blood eosinophilia peaks between 7 and 30 days after treatment, followed by a progressive return to normal levels thereafter (27). Despite this “acute” microfilaria-related blood eosinophilia, adult W. bancroti worms remained alive, as evaluated by ultrasonography until several months after treatment (34).

Fourth, the complex and intriguing phenomenon by which the adult worms escape eosinophil attack is illustrated by some well-defined clinical conditions, such as tropical pulmonary eosinophilia. This uncommon extra-lymphatic manifestation of lymphatic filariasis is characterized by peripheral eosinophilia, and by high titers of IgE and antifilarial IgG4 antibodies (35). However, in spite of pre-treatment blood eosinophilia of more than 20.000/mm^3^, living adult worms can be seen by ultrasound in intra-scrotal lymphatics, and these can remain unaffected by aggressive DEC treatment (12 mg/kg/day/30 days) (36). Thus, DEC is not 100% effective against adult worms and many of them are unaffected, even with increasing doses in both asymptomatic Mf-positive and Mf-negative individuals (26). Fifth, the so-called mixed reaction, in which alive and dead parasite share the same nest (19-21, 23, 26), demonstrates that some adult parasites are able to remain alive in spite of the strong inflammatory reaction directed against nearby damaged/dead worms. Of note, these mixed reaction have been observed in parasite nests from both DEC-treated and untreated patients. In our view, clarification of this remarkable phenomenon would help to unveil the mechanisms governing parasite resistance or susceptibility to the toxic products of eosinophils, antifilarial drugs, and natural conditions threatening the survival of adult worms. Sixth, in other filarial nematodes such as O. volvulus, there is ample evidence that DEC kills microfilaria and spares adult parasites, especially during the Mazzotti’s reaction when massive eosinophil recruitment and degranulation occur (30), which can threaten the patient’s life in cases of high parasite load. Localized lymphatic vessel inflammation with accumulation of eosinophils and subsequent degranulation was noted in a nodule formed as early as 7 days after beginning DEC treatment, when a clear granulomatous inflammatory reaction was not yet established. This finding explains why nodules can be detected by physical examination within seven day after the first dose of DEC intake (21, 26). Immunostained extra-cellular material and intact eosinophils were absent or scarce around remnants of *W. bancrofti* adult worms with calcification, a condition that supposedly “sterilizes” the parasite antigenic sources (25). Positive immunostaining was also observed in lesions 90 days after treatment, when non-calcified remnants of adult worms were barely discernable. The lifespan and the turnover of tissue-resident eosinophils are unknown. They usually undergo apoptosis after degranulation and are replaced by newly arrived cells; they arrive at their tissue destination prepared for immediate action or prolonged tissue survival (37). In such situation, they might have some effector activity in maintaining control of tissue repair and remodeling, angiogenesis, fibroblast activation and collagen production (12).

A remarkable finding in our material is the virtual absence of neutrophils in the tissue sections, even in earlier cases. This finding is relevant in view of the attention that has been paid to the *Wolbachia* endosymbiont bacterium present in most filarid nematodes and its link with neutrophilic infiltrates after parasite death (38). On the other hand, despite the absence of *Wolbachia* (39), dying *Loa loa* adult worms cause significant inflammation with initial neutrophil accumulation and suppuration, followed by granulomatous reaction (40). In addition, the number, size and cellular composition of granulomas in lymphatic vessels or renal lymph nodes did not differ after removal of Wolbachia in gerbils chronically infected with *B. pahangi*, indicating that *Wolbachia* may not play a pivotal role in the development of the lymphatic lesions (41). It is clear that additional studies are needed on *Wolbachia*-host interactions in the development of *W. bancrofti* granulomas and their impact on pathogenesis of adult worm-related chronic conditions.

Our current and previous studies in bancroftian filariasis give additional support to the concept that, as far as adult helminths are concerned, tissue eosinophilia appears to be triggered after the sudden release of antigens from parasites dying either spontaneously or following chemotherapy (42). The present study has limitations: (a) a relatively small number of cases; (b) the lack of a quatitative approach for histochemisty patterns; (c) as a retrospective investigation, it was not possible to perform plasma cytokine and eosinophil-derived proteins profiles. Thus similar investigations using more refined techniques would be welcome; (d) due to the rarity of palpable filarial nodule formation in females and children (24), specimens from these population were not included and it is not possible to extrapolate the results to these groups. In summary, based on a pioneering, integrated clinical, laboratorial and immunohistochemical investigation, our findings indicate that eosinophils *per se* are not responsible for the initiation of lethal damage to adult worms, as much as intrascrotal filarial granulomas are concerned. Rather, eosinophil influx and degranulation in DEC-related and non DEC-related bancrofitian granulomas likely represent a consequence of parasite death rather than its cause. However, eosinophils may play an important role in *postmortem* worm degradation as well as in granuloma evolution and modulation (12).
